# Immunomodulatory Effects of Nanoparticles on Skin Allergy

**DOI:** 10.1038/s41598-017-03729-2

**Published:** 2017-06-21

**Authors:** Samreen Jatana, Brian C. Palmer, Sarah J. Phelan, Lisa A. DeLouise

**Affiliations:** 1 Department of Biomedical Engineering, University of Rochester, Rochester, New York, USA; 2 Department of Toxicology, University of Rochester Medical Center, New York, USA; 3 Department of Dermatology, University of Rochester Medical Center, Rochester, New York, USA

## Abstract

In recent years there has been considerable effort to understand the interaction of nanomaterials with the skin. In this study we use an *in vivo* mouse model of allergic contact dermatitis to investigate how nanoparticles (NPs) may alter allergic responses in skin. We investigate a variety of NPs that vary in size, charge and composition. Results show that small (<200 nm) negative and neutral charged NPs exhibit an immunosuppressive effect but that positively charged NPs do not. Confocal imaging suggests positively charged NPs may penetrate skin to a lesser extent and thereby are less able interact with and alter the local immune responses. Interestingly, negatively charged silica (20 nm) NPs suppress allergic response to two chemically distinct sensitizers; 1-fluoro-2, 4-dinitrobenzene and 2-deoxyurushiol. Skin wiping and NP application time studies suggest that the immunomodulatory mechanism is not due solely to the blocking of sensitizer adduct formation in skin. Results suggest that NPs modulate early immune events that impact mast cell degranulation. Our study shows for the first time the potential to modulate the elicitation phase of the allergic response which depends on the NP charge and composition. These finding can be used to inform the design topical therapeutics to mitigate allergic responses in skin.

## Introduction

Biomedical applications of engineered nanoparticles (NPs) are rapidly expanding^1, 2^. The ability to manipulate the surface chemistry of NPs enables their targeted delivery to specific tissues and cells. NPs have been utilized for *in vivo* imaging^3^, to deliver antigen specifically to dendritic cells for more efficient vaccination and to deliver cytotoxic agents specifically to tumor cells^4, 5^. However, when NPs are administered *in vivo* they can act as foreign materials and induce unintended, off-target immune responses especially if they are not readily cleared from the body^6^. Understanding the immunomodulating effects of NPs is critical for the safe design and effective use of nano-therapeutics^7, 8^.

Current knowledge of the immunomodulatory properties of NPs comes largely from studies in the nanotoxicology field motivated by environmental health and safety concerns^9^. The immunomodulatory properties of NPs have been mostly studied by injection or exposing NPs to the respiratory tract using various animal models^10–13^. Fewer studies of NP skin interaction exist, even though simultaneous skin exposure to NPs and exogenous insults occur frequently^14–18^. Studies investigating the effect of NPs on skin allergy report that nano-TiO_2_ can exacerbate symptoms whereas positively charged SiO_2_ NPs had no impact^19–22^ and CaCO_3_ NPs reduced nickel induced skin allergy through an ion chelation effect^23^. Repeated topical application of silver NPs was also reported to reduce skin inflammation^24^. Reasons for these divergent results are unclear but likely reflect differences in the NP composition and skin penetration, the severity of the skin barrier defect, the allergen or dosing protocol used.

In this work, the effects that NPs have on mediating allergic responses in skin were examined using the well-established 1-fluoro-2, 4-dinitrobenzene (DNFB) contact hypersensitivity (CHS) mouse model of Allergic Contact Dermatitis (ACD)^25^. ACD is a delayed Type IV hypersensitivity response to sensitizers that contact skin and is characterized by pruritic erythema and blister formation at the site of inflammation depending on the severity of the response^26^. ACD sensitizers that contact skin trigger an adaptive immune response that is divided into two phases; the sensitization phase and the elicitation or challenge phase^27^. During sensitization an allergen (or chemical sensitizer) enters skin and becomes immunogenic. Chemical sensitizers are typically electrophiles that covalently interact with skin proteins to form an altered-self antigen that is recognized by the immune system^27, 28^. Antigen presenting cells (APC) in the skin uptake the immunogen, become activated, and migrate to the lymph node where they present antigen to naïve CD4+ T helper cells^29^. This leads to the development of antigen specific memory effector T cells^26^ such that on a second skin exposure (challenge) an inflammatory allergic reaction occurs^30^.

There are over 2800 chemicals commonly found in cosmetic products, in the environment and in the work place have the potential to cause ACD. These include for example, fragrances, formaldehyde and urushiol; the sensitizer in poison ivy^31–33^. ACD is a major occupational health hazard and accounts for almost 20% of work related exposures^34^. In contrast to all previous work investigating the effects of NPs on skin allergy, in this study we applied a single topical application of the DNFB sensitizer with or without NPs in the sensitization and/or challenge phases. We examined NPs that vary in composition, size, and surface charge using a consistent experimental protocol. In this study we demonstrate for the first time that small (<200 nm) negatively charged NPs exhibit immunosuppressive effects whereas positively charged and poorly soluble NPs prone to agglomeration had either no effect or exacerbated the allergic response. Our results suggest the potential to design NP-based topical lotions that can prevent or treat allergic skin inflammatory disorders.

## Results and Discussion

**Figure 1:**
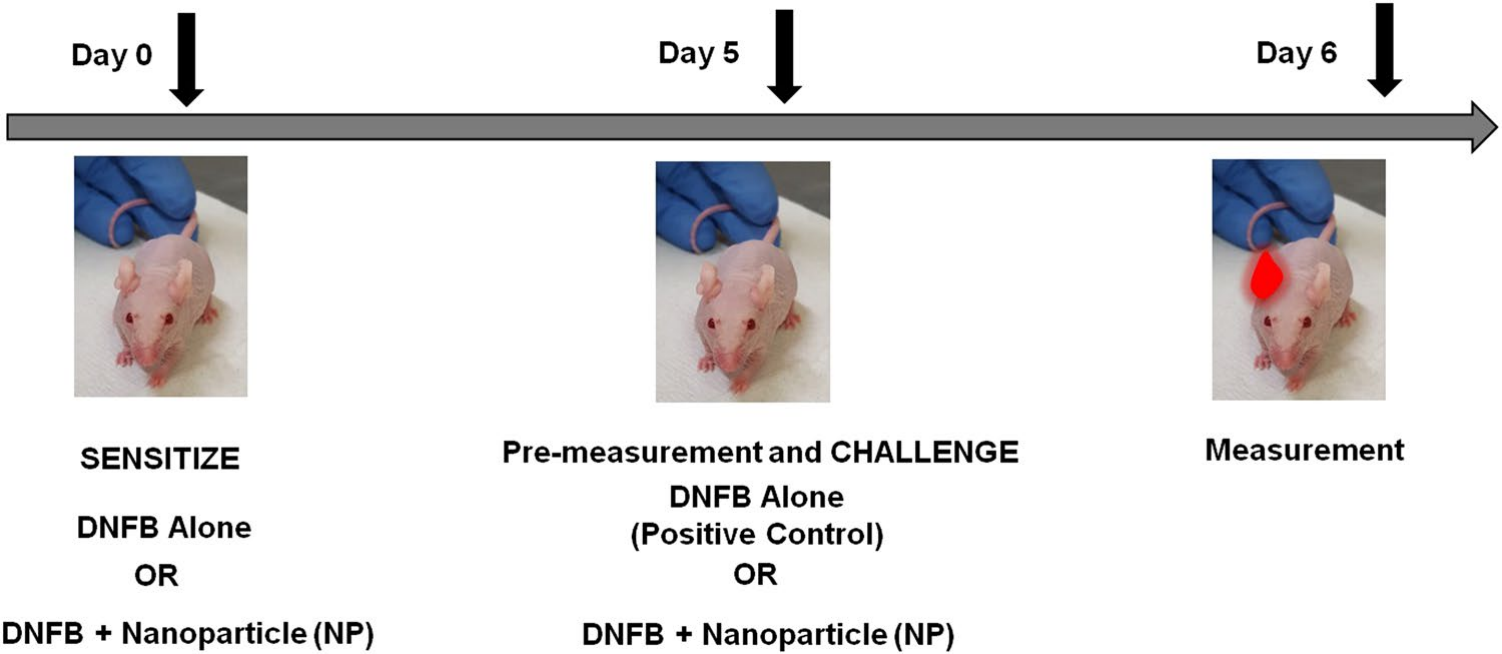
Protocol for testing the effect of NPs on the sensitization and challenge phases in the contact hypersensitivity (CHS) mouse model using C57BL/6 hairless mice. The mice were sensitized on day 0 with 1) 0.05% DNFB alone, 2) 0.05% DNFB with negatively charged GSH coated QD, or 3) 0.05% DNFB with positively charged PEI coated QD, in acetone/olive oil medium on the dorsal back. On day 5 both the right and left ears were pre-measured before the challenge. The ears were challenged with 1) 0.2% DNFB alone, 2) 0.2% DNFB along with combinations of differently charged QD (QSH-QD negative charge, PEI-QD are positive charge, mPEG-QD neutral and lipophilic QD), or 0.2% DNFB along with combinations of other NPs that are commercially available (silica NP, gold NP, Ag NP, carbon nanotubes and titanium dioxide). On day 6, 24 hours after the challenge, the ears were measured and compared to the baseline pre-measurements. During the course of treatment, the right ears always served as the 0.2% DNFB alone treated ear and the left ears were either vehicle control or 0.2% DNFB + NP treated ears. Due to variability in animal reactivity to DNFB, the swelling of DNFB + NP co-treated ears was always compared to the swelling of control DNFB ears within the same mouse.

**Figure 2:**
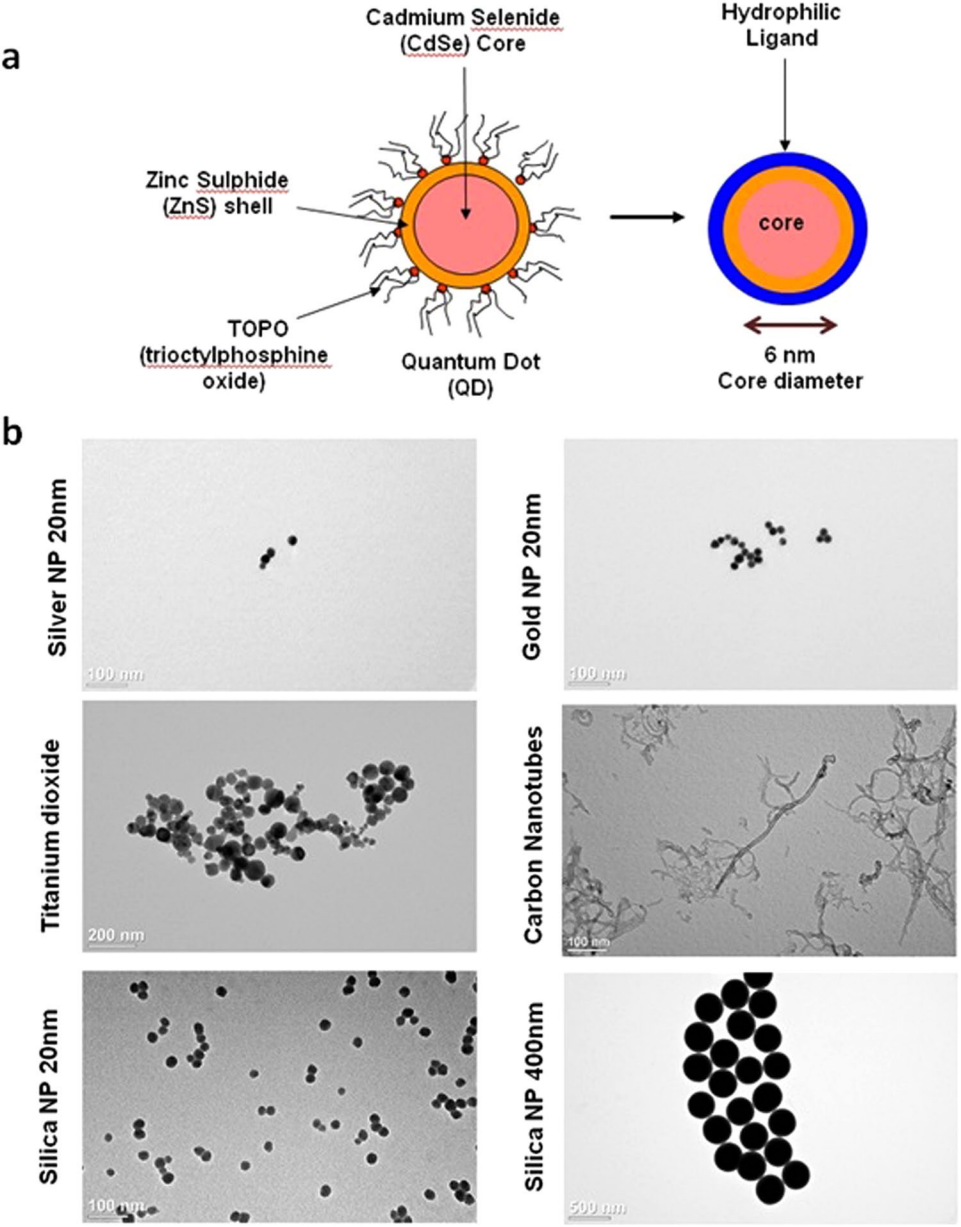
Quantum dot preparation and nanoparticle characterization. (**a**) Schematic demonstrating the ligand exchange method used to alter the surface coating on commercial QDs. Ligand exchange methods are used to coat QD with hydrophilic ligands to make them water soluble and to alter surface charge. (**b**) Characterization of NPs using Transmission Electron Microscopy (TEM). TEM was used to qualitatively evaluate NP size and agglomeration of various NPs purchased from vendors.

In this study, we examined the effect of a wide variety of topically applied NPs (Supplementary Table 1 **)** to modulate the CHS response to DNFB using the protocol outlined in Fig. 1. NPs included quantum dots (QDs) that were coated with hydrophilic ligands using an in-house ligand exchange protocol (Fig. 2a) and other NPs purchased from vendors and used directly. Their characteristics including surface charge, size and polydispersity using were quantified using a Malvern Zetasizer and Transmission Electron Microscopy (TEM) (Fig. 2b, Supplementary Tables 2 and 3). The mice used in this study were fully immunocompetent hairless SKH-1 mice backcrossed into a C57BL/6 background and >5 months old which we found was important to obtain consistent allergic response measurements. Animal experiments were approved by the University Committee on Animal Resources (UCAR) at the University of Rochester Medical Center (#100360/2010–024). In a typical CHS study, the mice are topically sensitized on Day 0. Five days after sensitization, the mice are challenged with DNFB on the ears and the ear swelling response is measured using digital calipers. In this study, we challenged the mice with either DNFB alone, NP alone and/or co-challenged using a combination of NP plus DNFB. Sensitization was done with 0.05% DNFB and challenge with 0.2% DNFB mixed in 4:1 acetone/olive oil vehicle alone or in combination with NPs. It is common in the literature to sensitize with 0.5% DNFB but this caused severe tissue burns in our mice and lowering the sensitization to 0.05% DNFB produced an equivalent ear swelling response upon challenge (Supplementary Figures S1 and S2).

**Figure 3:**
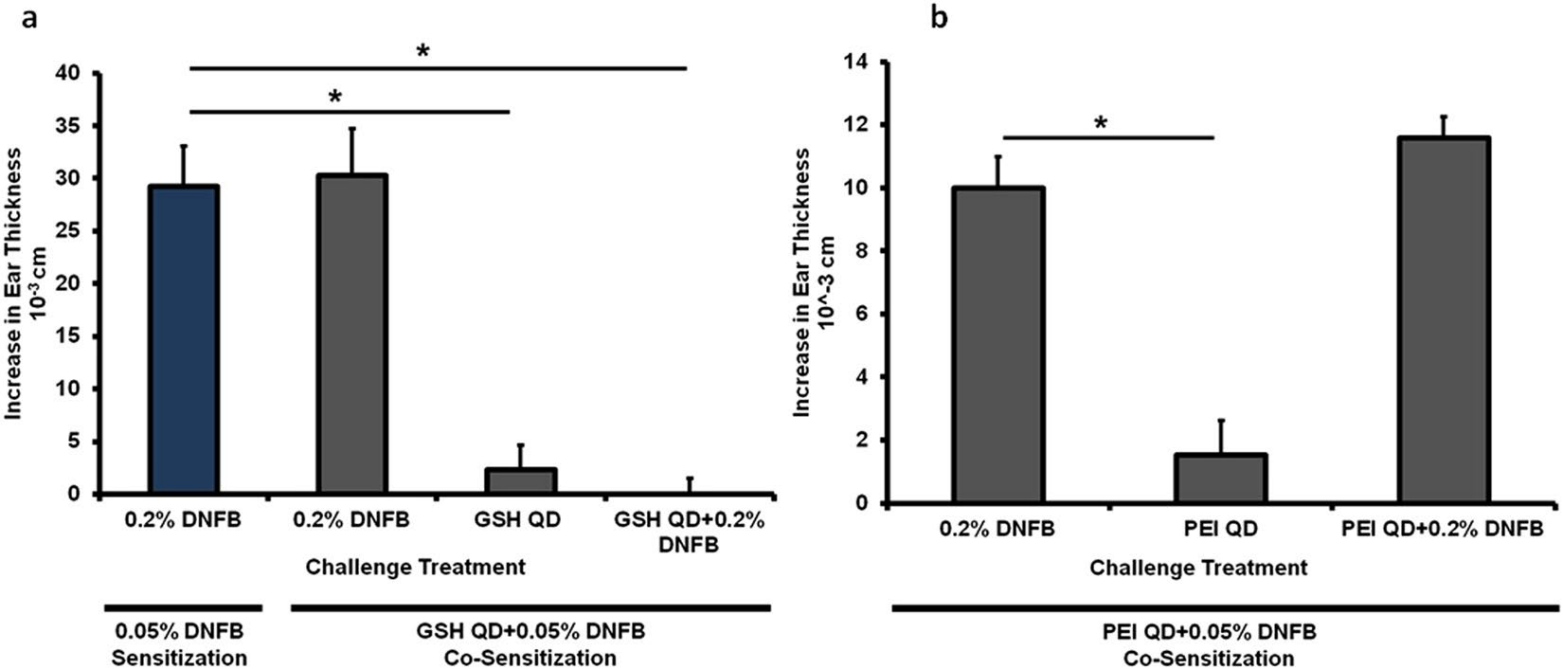
Co-sensitization with 0.05% DNFB + GSH QD and 0.05% DNFB + PEI QD. (**a**) Mice were co-sensitized to either 0.05% DNFB alone or 0.05% DNFB + GSH-QD. Various combinations were tested in the challenge phase. The co-sensitized mice exhibited a normal ear swelling response when challenged with 0.2% DNFB alone which was comparable to the ear swelling levels measured on mice sensitized with only 0.05% DNFB (blue bar, expected swelling response). Mice did not exhibit any ear swelling response to GSH-QD alone indicating that they were not sensitized to GSH-QD. When GSH-QD were combined with 0.2% DNFB in the challenge phase, the ear swelling response was reduced. These mice were sensitized to DNFB, but the combination with the nanoparticle inhibited the ear swelling response in the challenge phase. (**b**) Mice were co-sensitized to 0.05% DNFB + PEI-QD. They exhibited a normal ear swelling response to both 0.2% DNFB alone and co-challenge with DNFB + PEI-QD indicating that the mice were sensitized to DNFB and that PEI-QD did not suppress the inflammation in the challenge phase. No ear swelling response was observed to mice challenged with PEI-QD alone again indicating that the animals were not sensitized to PEI-QD. Mean ± SEM, *p < 0.05, Student’s 2-tailed t-Test, paired with unequal variances, N = 3–4.

Initially, we examined the effects of topically applied CdSe/ZnS core/shell quantum dots (QDs) with various surface coatings. The QDs were prepared using previously described methods^35^ and characterized as summarized in the Methods Section and Supplementary Table 2, respectively. First, we tested the effect of negatively charge glutathione-coated QDs (GSH-QDs) mixed with DNFB for sensitization. Details on the preparation of QD mixtures used in CHS experiments are given in Supplementary Table 4. Five days post sensitization the mice were challenged on their ears with DNFB alone, with GSH-QDs alone or they were co-challenged by applying DNFB + GSH-QDs simultaneously onto the ear. The results in Fig. 3a show that co-sensitized mice challenged with DNFB alone exhibited an expected magnitude ear swelling response. This suggests that the presence of GSH-QDs during sensitization did not alter hapten formation or the generation of effector T cells. Mice challenged with GSH-QDs alone did not exhibit an ear swelling response indicating that the mice were not sensitized to the GSH-QDs even in the presence of the powerful DNFB sensitizer. Interestingly, when the mice were co-challenged with GSH-QD + DNFB simultaneously, the ear swelling response was reduced even though the DNFB was applied in an estimated >10^5^ molar excess to the GSH-QDs. Importantly, an expected magnitude ear swelling response was measured when the mice were co-challenged with DNFB mixed with free glutathione (Supplementary Figure S3) suggesting that the immunosuppressive effect was related to the QDs. Next, we tested the effect of positively charged polyethylenimine quantum dots (PEI-QDs) mixed with DNFB on sensitization. An expected magnitude ear swelling response was similarly measured when these mice were challenged with DNFB alone and no swelling was observed when they were challenged with PEI-QD (positively charged) alone **(**Fig. 3b). However, when the mice were co-challenged with the PEI-QD + DNFB a significant ear-swelling response was measured (Fig. 3b) which is in contrast to the GSH-QDs which suppressed ear swelling.

**Figure 4:**
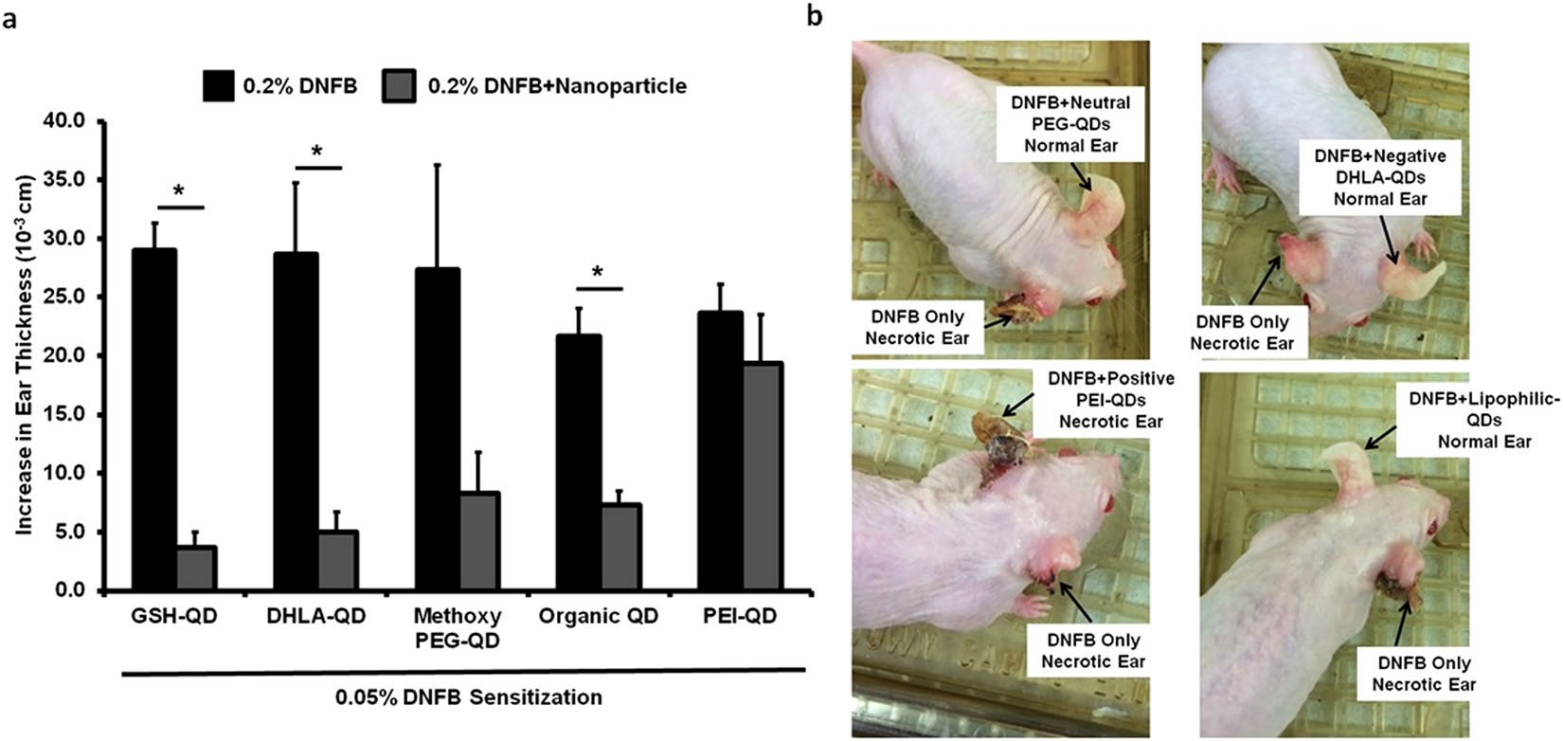
Co-challenge experiments with Methoxy PEG-QD, DHLA-QD, organic (lipophilic) QDs and PEI-QD. (**a**) Mice were sensitized to 0.05% DNFB. In the challenge phase, the right ear was exposed to 0.2% DNFB alone and the left ear was co-challenged with 0.2% DNFB + QD combinations. GSH-QD (negative charge), DHLA-QD (negative charge), Methoxy PEG-QD (neutral charge) and organic QD all inhibited the ear swelling response (p = 0.01, p = 0.02, p = 0.07 and p = 0.005 respectively). PEI-QD with a positive charge did not inhibit the ear swelling response (p = 0.42). Mean ± SEM, *p < 0.05, 2 tailed t-Test, paired with unequal variances, N = 3. (**b**) These mice were followed for a month after the challenge phase. The right ear treated with 0.2% DNFB forms scabs 72 hours after challenge and the ear necrotic after 4 weeks. The groups in which the QD inhibited the ear swelling response, the left ear is intact after a month. PEI-QD did not inhibit the swelling response so the left ear is necrotic as shown in the gross image.

To further investigate this, we tested QDs with other surface coatings and charges in the challenge phase (Supplementary Methods and Supplementary Tables 2 and 4). Mice were sensitized with DNFB only and challenged with DNFB alone or co-challenged with the DNFB + QDs. Results (Fig. 4a) show that negatively charged DHLA-QDs, neutral charged methoxy-PEG-QDs and the organic QDs (trioctylphosphine oxide coated) all reduced the ear swelling response whereas again, the positively charge PEI-QDs did not. These mice were followed for one month post challenge (Fig. 4b). We observed that the ears treated with DNFB alone formed scabs at ~72 hours post-challenge and they became necrotic after 4 weeks. The ears treated with QDs that reduced the swelling response remained healthy after 4 weeks whereas the ears co-challenged with PEI-QDs also became necrotic. These results suggest that the mechanism inhibiting the ear swelling response may depend on QD charge; possibly by affecting either the bioavailability of DNFB in the skin or the ability of the QDs to interact with skin cells to alter the immune response.

**Figure 5:**
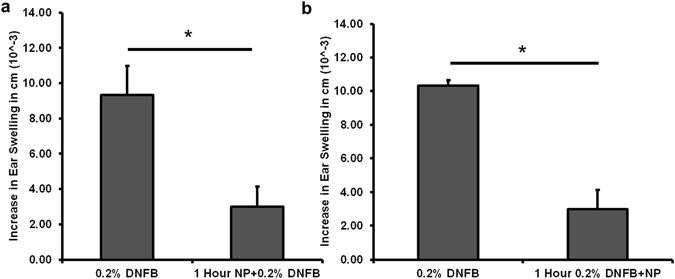
GSH-QDs applied in a step-wise manner, before or after DNFB application. (**a**) We applied the GSH-QDs to the mouse ear for 1 hour, then wiped the majority of the GSH-QDs off with a PBS soaked cotton swab before the DNFB challenge dose was applied. DNFB application to the wiped ear has a reduced swelling response (1 Hour NP + 0.2% DNFB). (**b**) DNFB was applied to the ear first for 1 hour followed by GSH-QD application. The ear swelling response was reduced. Mean ± SEM, *p < 0.05, Student’s 2-tailed t-Test, paired with unequal variances, N = 3–4.

In an effort to test whether the GSH-QDs inhibitory ear swelling response was due to blocking DNFB skin penetration we administered the GSH-QDs in a stepwise manner. Five days post-sensitization we applied the GSH-QDs to the mouse ear for 1 hour and then wiped the ears with a cotton swab soaked in PBS to remove excess GSH-QDs. After wiping, the DNFB challenge dose was applied. Ear thickness measurements made at the 24 hour time point showed a reduced swelling response (Fig. 5a). Since it is likely that after wiping, some GSH-QDs were retained in hair follicles, skin furrows and/or outer layers of the epidermis, we altered the protocol to first challenge the ear with DNFB alone and then 1 hour later the GSH-QDs were applied. Results consistently showed a reduced ear swelling response measured 24 hour post challenge (Fig. 5b). The step-wise exposure studies, along with the fact that DNFB is co-administered in ~1 × 10^5^ molar excess to GSH-QDs, suggests that the reduced ear swelling is not due solely to blocking DNFB adduct formation in skin.

Scanning confocal microscopy was used investigate the potential for QDs to interact with skin cells as previously described^36^. Results show that the PEI-QDs tend to concentrate more in the outermost skin layers but follicular accumulation is evident (Supplemental Figure S4). Analysis of the image stacks shows that the GSH-QDs, the methoxy-PEG-QDs and the organic QDs all penetrate the epidermis to a greater extent than the PEI-QDs especially when examining the magnitude of the QD fluorescence signal at 10 microns below the stratum corneum (Supplemental Figure S5). Penetration of positively charged QDs into and beyond the stratum corneum is likely hindered by agglomeration and/or electrostatic attraction to the negatively charged skin surface which may limit their potential to interact with and modulate skin cell responses.

**Figure 6:**
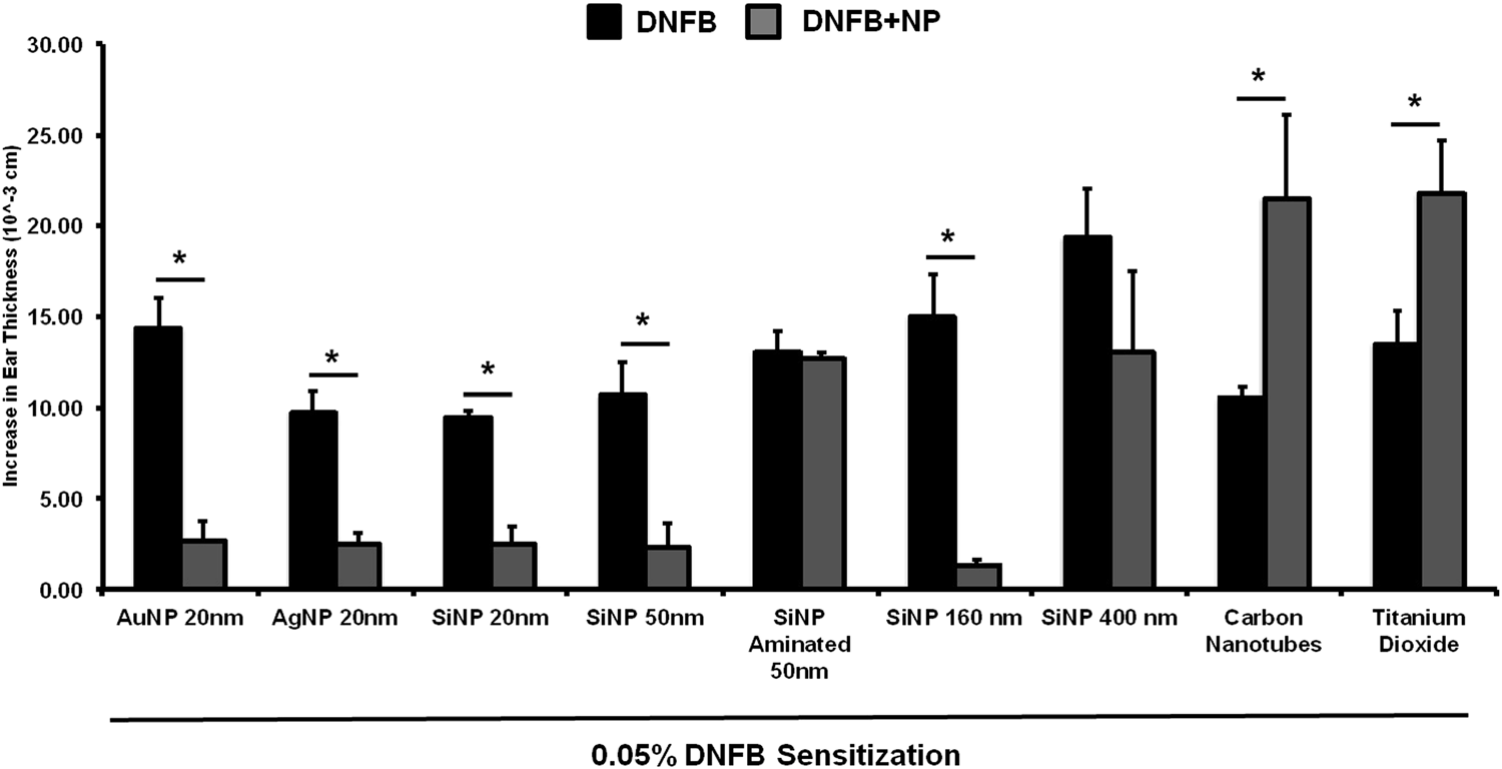
Co-challenge experiments with different NP types in mice sensitized to DNFB. Mice were sensitized to 0.05% DNFB alone. In the challenge phase, the right ear was exposed to 0.2% DNFB and the left ear was co-challenged 0.2% DNFB + NP combination. AuNP (20 nm), Silica NP (20 nm, 50 nm, 160 nm) and AgNP (20 nm) reduced the ear swelling response. Aminated Silica NP (positive charge) had no effect.  Carbon nanotubes (CNT) and Titanium dioxide (TiO_2_) increased the ear swelling response significantly over the control. Silica NP (400 nm) trended to reduce the swelling response but it was not significant compared to the control ear. Mean ± SEM, *p < 0.05, 2 Student’s tailed t-Test, paired with unequal variances, N = 3–4.

We further examined the effect of NP composition, charge and size on modulating allergic ear swelling response. The NPs tested included citrated gold (20 nm), citrated silver (20 nm), and hydroxylated silica (20 nm, 50 nm, 160 nm, 400 nm) which are all negatively charged (Fig. 2b and Supplementary Tables 1 and 3). Results (Fig. 6) indicate that these NPs (except 400 nm SiNPs) significantly reduced the ear swelling response compared to the DNFB treated ear alone (p < 0.05). We also tested positively charged aminated silica (50 nm) which did not suppress the swelling response further suggesting that NP charge is crucial in mitigating the immunosuppressive response. Furthermore, we tested poorly soluble carbon nanotubes (CNT) and titanium dioxide (TiO_2_) which both exacerbated the swelling response. The above results were confirmed by H&E stained ear sections (Supplementary Figure S6 **)**. The TiO_2_ response is not unexpected, as it is widely known that TiO_2_ NPs agglomerate on the skin surface and a recent study reported that topical application of nano-TiO_2_ particles to mouse ears induced an irritation swelling response in a dose dependent manner^37^.

**Figure 7:**
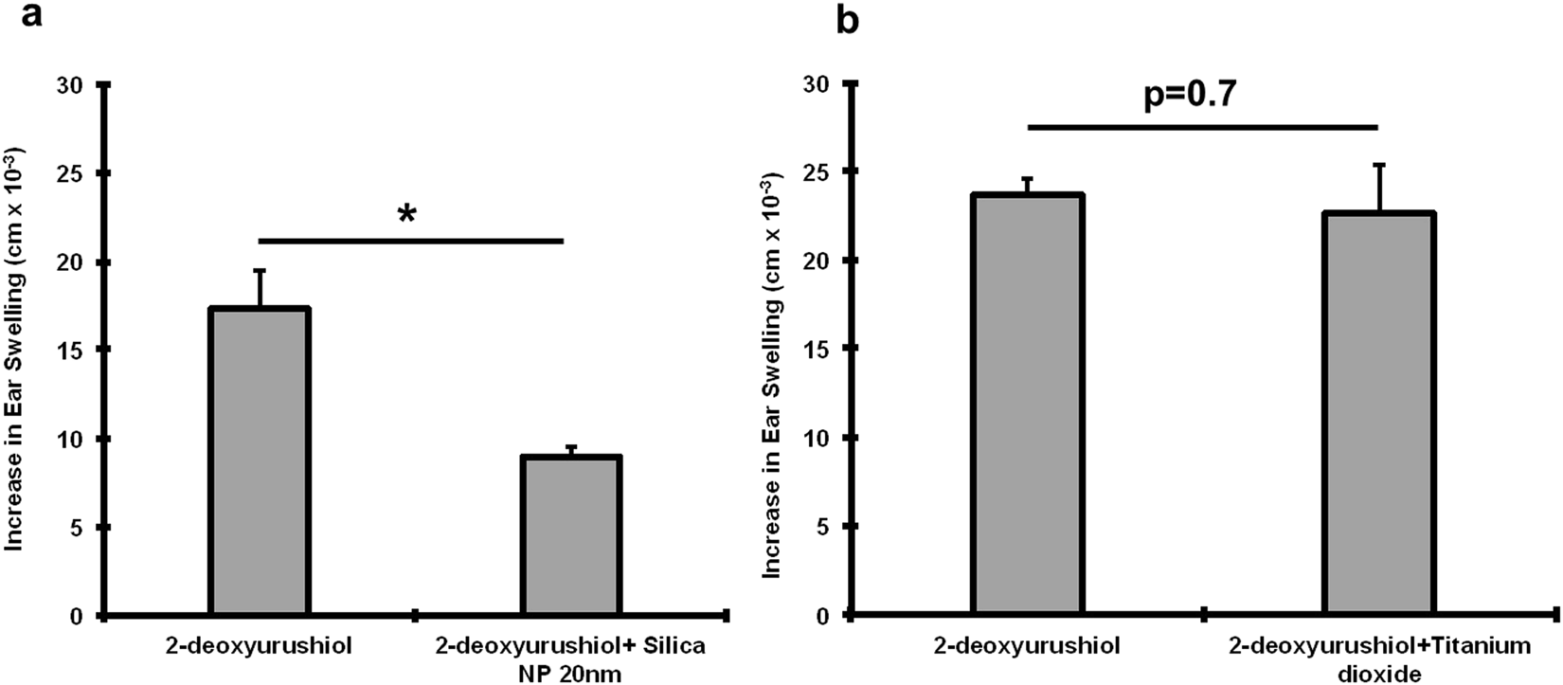
Co-challenge experiment with silica NP and TiO_2_ NP sensitized to 2-deoxy urushiol. Mice were sensitized with 15% 2-deoxyurushiol in an acetone vehicle. The right ears were challenged with 15% 2-deoxyurushiol alone and the left ears were co-challenged with 15% 2-deoxyurushiol and (**a**) silica NP (20 nm) or (**b**) TiO_2_ mixed in an acetone/olive oil vehicle. The silica NP inhibited the ear swelling response to 2-deoxyurushiol. Mean ± SEM, *p < 0.05, 2 Student’s tailed t-Test, paired with unequal variances, N = 3–4

**Figure 8:**
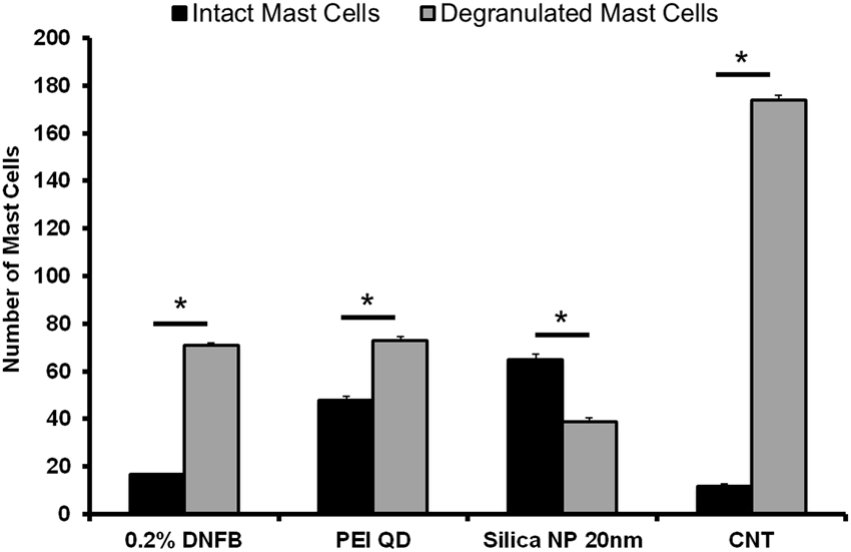
Mast cells quantified in the ear tissue by IHC using the Geimsa stain. The number of degranulated MCs was significantly lower in the silica NP (20 nm) treatment group compared to PEI-QD and CNT treated ears co-challenged ear. MCs were counted in 10 fields of view at 40X for 3 independent mouse skin samples. Mean ± SEM, *p < 0.05, 2-tailed Student’s t-Test, paired, with respect to intact mast cells in each group. Error Bars: Standard Dev.

**Figure 9:**
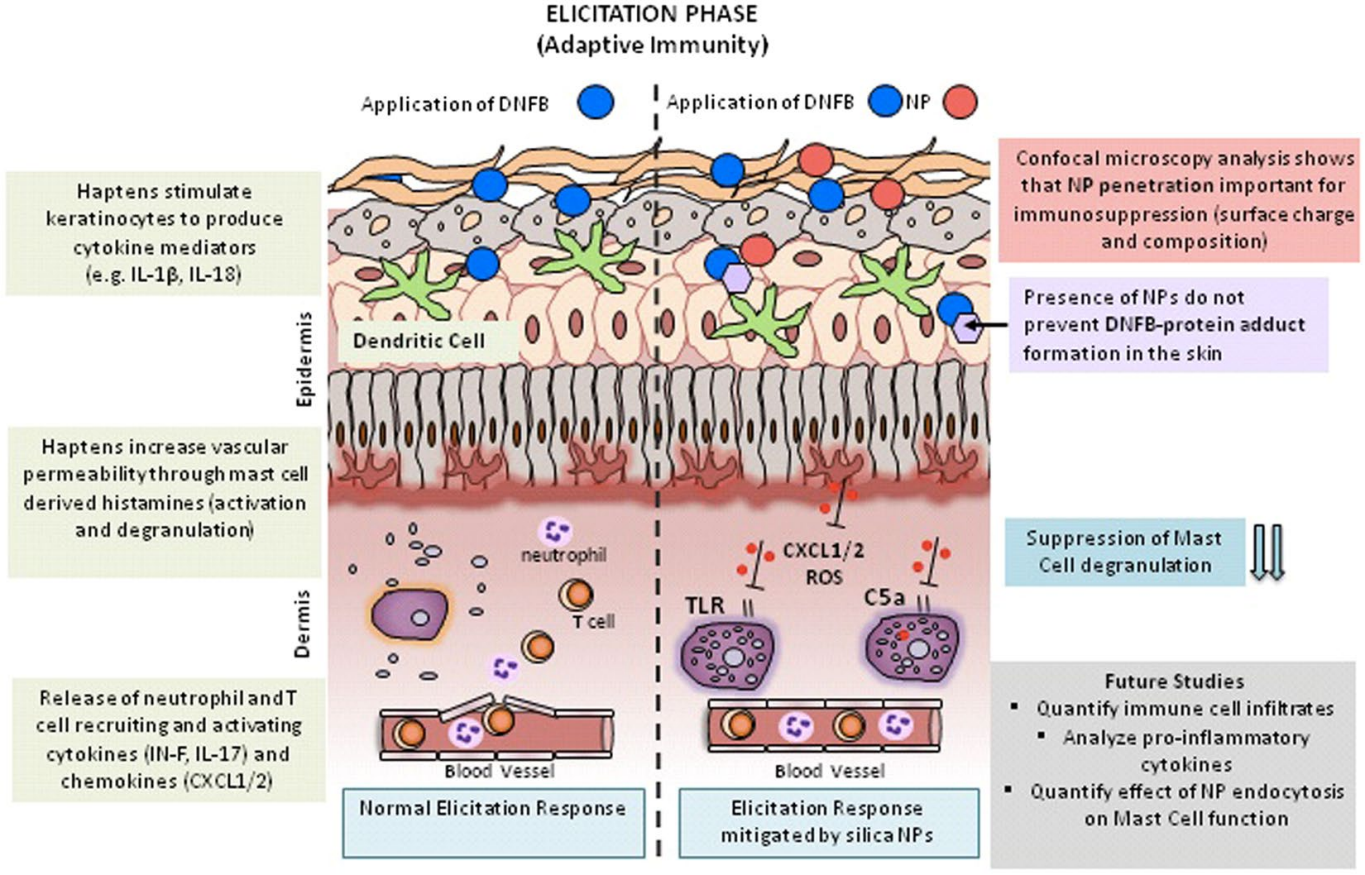
Schematic summary of findings, future directions and postulated mechanisms of action of NPs in the elicitation phase (adaptive and innate immunity). The illustration contrasts the difference between a normal elicitation response and how negatively charged NPs <200 nm may possibly modulate the immune responses when co-challenged with a T_H_1 sensitizer like DNFB. In a normal elicitation response the sensitizer combines with skin proteins to form an immunogenic hapten that stimulates keratinocytes to produce cytokine proinflammatory mediators like ROS, IL-1β and IL-18. These signals activate vascular endothelial cells that guide immune cells to the tissue. Early cascade of events (2–4 hours) involves mast cell degranulation, histamine release and secretion of neutrophil recruiting chemokines like CXCL1 and CXCL2. Between 12–24 hours of elicitation, antigen-specific memory T cells are recruited to the tissue and are activated by local antigen carrying dendritic cells. This leads to production of IFN-γ and IL-17, which further activates tissue resident T cells and causes a full blown allergic response. Our results indicate that NP charge, composition and presence in the tissue modulates the ear swelling response in mice by possibly mitigating early immune events including mast cell degranulation and subsequent recruitment of other immune cells. NPs may modulate epidermal derived signals that inhibit complement or TLR receptor signaling. NPs may also be endocytosed by mast cells that can affect their function.

Collectively, these results indicate that NP composition, surface charge and primary particle size and/or agglomeration state are important factors for modulating the ear swelling responses in the DNFB CHS mouse model. It is important to note that in this preliminary study the NPs were applied at a constant mass (Supplemental Table 4). This means that we did not control for particle number or the NP surface area applied. However, these are not confounding factors for comparing the positively (aminated) and negatively (hydoxylated) charged 50 nm silica NPs or the positively (PEI) and negatively (GSH) charged QDs which clearly show that negatively charged, but not positively charged NPs reduce the ear swelling response. In on-going studies we are investigating a silica size series to determine if the ear swelling suppression correlates with NP size, particle number or surface area. Although many of the NPs tested here are already used in consumer products, silica is an ideal material to investigate further due to is biocompatibility, degradation to nontoxic silicic acid and its wide use in drug delivery systems^38, 39^. In preliminary testing we observed that negatively charged silica (20 nm) also suppressed the allergic ear swelling response to 2-deoxyurushiol, a chemical analogue of urushiol but TiO_2_ NPs did not (Fig. 7 **)**. Future studies will seek to refine the time window (before and after) that NPs can be applied to skin relative to sensitizer in the challenge phase which is relevant for clinical translation of our findings. Although there are architectural differences between mouse and human skin, *ex vivo* studies suggest that the interaction of NPs with mouse and human skin follow similar trends^36^. It is equally important to examine the underlying immunomodulatory mechanisms. Our initial study of ear histology sections suggests a positive correlation between the immunomodulatory effect of NPs on ear thickness and mast cell degranulation **(**Fig. 8). We find that the number of degranulated mast cells is significantly lower in the SiNP (20 nm) treatment group compared to PEI-QD and CNT co-challenged ears. Mast cells are tissue resident cells that are key promoters of contact allergy^40^. Mast cell activation and degranulation release histamines and other cytokine mediators that are essential for the early response in the elicitation phase^41^. They control recruitment of other immune cells into skin^42, 43^ and studies show that MC deficient mice exhibit dramatically reduced CHS response^40^. Hence, in future work will investigate the effect of NPs on the mediators of mast cell activation and degranulation that include engagement of Toll-like receptors (TLRs), high affinity IgE Fc-receptors and complement-receptors. However, in our CHS model, DNFB is a T_H_1 hapten so it is likely that degranulation by IgE crosslinking of Fc-receptors is a primary mechanism. It is of interest to note, that the magnitudes of the changes in ear thickness **(**Fig. 6) do not correlate exactly with mast cell degranulation (Fig. 8) suggesting that other immune cells may be involved. Hence, we plan to quantify the effect of NPs on skin cellular infiltrates including T cells (CD4+, CD8+), MHCII+ cells, Langerhans cells, mast cells, basophiles and neutrophils as well as the modulation in cytokine levels in the skin and lymph nodes during the sensitization and challenge phases. Our results and possible mechanisms in the CHS elicitation response influenced by NPs are summarized in Fig. 9.

In summary, this paper investigates the immunomodulatory effects of variety of NPs that vary in size, charge and composition on skin allergy using the well-established DNFB CHS mouse model of ACD and a consistent protocol with the NPs applied at an equivalent mass dose. We have discovered that some NPs, particularly small (<200 nm) and negatively charged NPs exhibit immunosuppressive effects whereas similarly sized positively charge NPs do not and other NPs prone to agglomeration exacerbate allergic symptoms when co-challenged with DNFB. Interestingly, silica (20 nm) NPs suppress ear swelling response to two chemically distinct sensitizers; DNFB and 2-deoxyurushiol. Moreover, wiping off excess QD NPs prior to DNFB challenge or application of QDs 1 hour post DNFB challenge suppress the ear swelling response, suggesting that the immunomodulatory mechanism may not be due solely to the blocking of DNFB adduct formation in skin. The confocal data and free glutathione studies suggests NP skin penetration is important for modulating the CHS response. In future studies we will examine the cellular and molecular level mechanisms underlying the observed immune modulating NP effects reported here. We will test the hypothesis that NP presence in the skin modulate early (hours) innate immune events that impact mast cell degranulation which orchestrate the longer term (24 hr) T cell responses in skin.

## Methods

### Quantum Dot (QD) functionalization and NP characterization

Commercially available Cadmium Selenide-Zinc Sulphide (CdSe-ZnS, 5.8 nm core diameter, 600–620 nm emission peak) core-shell nanocrystals dissolved in toluene and capped with trioctylphosphine oxide (TOPO) were modified using a ligand exchange protocol to render them water-soluble (Fig. 2a). QDs were coated with Glutathione (GSH, negative surface charge), polyethylenimine (PEI, positive surface charge), dihydrolipoic acid (DHLA, negative surface charge) and methoxy polyethylene glycol (PEG, neutral surface charge) to alter the surface charge. The concentration of the sample was determined by measuring the UV-Vis absorbance on a Nanodrop spectrophotometer at the first exciton using Lambert-Beer’s Law. The Malvern Zetasizer Nano ZS was used to determine the hydrodynamic diameter by light scattering and surface charge by zeta potential measurements in distilled water (pH = 6.7). The QD properties have been summarized in Supplementary Tables 1, 2 and 3. Transmission Electron Microscopy (TEM) was used to qualitatively evaluate NP size and agglomeration (Fig. 2b).

### *In vivo* nanoparticle sensitization and challenge experiments

All mice used in this study are hairless C57BL/6, which contain a genetic mutation that causes alopecia to develop after the first hair follicle maturation. This phenotype is preferred for topical exposures, since the use of other breeds necessitates hair removal, which may cause a barrier defect in the epidermis and hence facilitate NP penetration. The mice do not have hair but their hair follicles are intact. Mice were either male or female with ages that range from 5–6 months old. Animal experiments were approved by the University Committee on Animal Resources (UCAR#2010–024/100360) at the University of Rochester Medical Center. All methods were performed in accordance with the relevant guidelines and regulations. The mice were housed in standard cages, up to four mice per cage, with access to food and water *ad libitum*. However, after sensitization, the mice were housed individually to prevent grooming. The schematic for CHS protocol is outlined in Fig. 1. Briefly, mice were sensitized on Day 0 using 0.05% dinitrofluorobenzene (DNFB), a common T_H_1 chemical hapten, in an acetone/olive oil vehicle (4:1 acetone:olive oil volume ratio). This dose was titrated down from 0.5% to avoid skin irritation (Supplemental Figures S1 **)**. The sensitization dose (30 µl) was pipetted on dorsal side right above the tail region. Five days post-sensitization, both the right and left ears were pre-measured using digital calipers prior to the application of the challenge dose. A challenge dose of 20 µl, 0.2% DNFB in 4:1 acetone:olive oil vehicle, was pipetted on the right ear (10 µl on each the dorsal and ventral side of the ear). The left ear was challenged using the vehicle alone (20 µl). 24 hours post-challenge, the ear swelling response was measured using calipers. The mice were euthanized via CO_2_ asphyxiation and the ear tissue was collected and stored at −80 °C for staining. In the co-sensitization study, GSH-QDs and PEI-QDs were mixed with 0.05% DNFB in acetone/olive oil vehicle in a fixed concentration and applied on the dorsal side on Day 0. In the co-challenge study, the different NP types were mixed with 0.2% DNFB and applied on the left ear, the right ear was challenged using 0.2% DNFB alone (Supplementary Tables 1 and 4). In both the co-sensitization and co-challenge study, the DNFB was in molar excess of the NP concentration. For the 2-deoxyurushiol studies, 100 μl of 15% 2-deoxyurushiol in an acetone vehicle was pipetted onto the dorsal side of the mouse just above the tail for the sensitization. After 5 days, 20 μl of the 15% 2-deoxyurushiol solution was pipetted onto the ear (10 μl on both the dorsal and ventral sides). One ear was treated with 2-deoxyurushiol alone and the other received both 2-deoxyurushiol and 20 nm silica nanoparticles (20 µg total dose).

### Quantification of the ear swelling response

Both right and left ear thickness was measured using digital calipers on Day 5 before the application of the challenge dose and recorded as the pre-challenge ear thickness. 24 hours after challenge, the swelling response was measured and recorded as the post-challenge ear thickness. Data are expressed as follows: change in ear thickness = (post-challenge ear thickness)−(pre-challenge ear thickness). To examine the ear swelling via a secondary qualitative method, 5 µm frozen sections of the ears were cut using a Thermo Scientific Cryotome FE. These sections were placed on glass slides and stained with hematoxylin and eosin dye using standard procedures. General tissue histology and cell infiltrates were observed using a Nikon Eclipse E800 bright field microscope.

### *Ex vivo* mouse skin QD exposure and quantification using confocal microscopy

To quantify QD penetration through mouse skin, all QD types were topically applied on mouse skin (4 cm^2^ area) in the acetone/olive vehicle for a duration of 24 hours at room temperature in an *ex vivo* set up. Skin samples were placed in a petri dish on gauze soaked in culture media to keep the skin hydrated during the topical exposure. After the 24 hour exposure, the residual vehicle was wiped off the stratum corneum and the skin samples were imaged using confocal laser scanning microscopy (CLSM) from 0–40 µm into the viable epidermis. The stacks collected using CLSM were quantified for QD presence using the histogram function on Image J analysis software.

### Confocal Imaging: Analysis of Stacks

The system was adjusted so negligible autofluorescence was observed at the QD emission peak (605 nm) in the control mouse skin. Images obtained using CLSM were processed using Image J Analysis software (NIH, version 1.48). Each image (8 bit) was split into 3 channels, the red channel (QDs) was retained for analysis and the pixel information was extracted using the histogram function. A high threshold for fluorescence signal between 220–255 on the grey scale representing QDs was set on Image J for the purpose of quantification. The pixel number was averaged to obtain relative intensity of the QDs in each individual image between the depths of 0–40 µm. A cut off depth of 40 µm for imaging was set to quantify penetration differences into viable epidermis in the different treatment groups.

### Histology

Frozen ear tissue was embedded in O.C.T compound (Fischer HealthcareTM Tissue PlusTM) cryosectioning. The tissues were sectioned into 5 µm thick sections using Thermo Scientific Cryotome FE. Geimsa stain (Sigma Aldrich, Catalog No: GS) was used to analyze mast cells. The Geimsa stain nuclei in varying shades of purple and the cytoplasm is stained blue to light pink. Eosinophils and red blood cells are stained shades of pink and bright orange, whereas mast cell granules are stained purple. The sections were imaged at 40X magnification on a Nikon Eclipse E800 microscope and RT3 camera/spot advanced software (version 4.6). With the help of a dermatopathologist (Dr. Glynis Scott, URMC-Department of Dermatology), the number of mast cells (intact vs. de-granulated) were counted in each section. The sections were also stained with hematoxylin and eosin stain (H&E) using standard procedures and the sections were imaged at 40X magnification (Nikon Eclipse E800).

### Instruments and Software

Digital calipers manufactured by Kroeplin (#C11OT) were used to measure the mouse ears. Cryotome FE (Thermoscientific) was used to section the mouse ear tissue for histology. Zetasizer Nano (Malvern Instruments) and Nanodrop was used to quantify the size, charge and concentration of the QD samples. Nikon E800 microscope was used to image the histology sections and RT3 camera/spot advanced software (version 4.6) was used to acquire the images included in the supplementary data.

### Statistical Analysis

Two-tailed Student’s t-test, unpaired with unequal variances, was used to compare penetration differences between different QD applications in the *ex vivo* penetration study (N = 5). 2-tailed Student’s t-test, paired with unequal variances, was used to compare the ear swelling measurements. Data are represented as change in swelling response compared to the pre-measurement value before the challenge (baseline thickness of the ear). P < 0.05 was considered to be significant. Error bars represent standard error of mean (SEM). The number of mice used in each experiment has been mentioned under individual plots.

## Electronic supplementary material

Supplementary Data File
