# Supplementary material for: Immunomodulatory Effects of Nanoparticles on Skin Allergy
Source: Sci Rep. 2017 Jun 21;7:3979. doi: 10.1038/s41598-017-03729-2 (PMC5479793; doi:10.1038/s41598-017-03729-2)
Supplement: Supplementary file 1 — Supplementary Data File [file 41598_2017_3729_MOESM1_ESM.pdf]

Supplementary Methods, Tables and Figures

Immunomodulatory Effects of Nanoparticles on Skin Allergy

Samreen Jatana[a], Brian C. Palmer[b], Sarah Phelan[b], Lisa A. DeLouise[a,c]

[a] Department of Biomedical Engineering, University of Rochester, Rochester, New York, USA.
[b] Department of Toxicology, University of Rochester Medical Center, New York, USA
[c] Department of Dermatology, University of Rochester Medical Center, Rochester, New York, USA.

Lisa A. DeLouise
University of Rochester Medical Center
School of Medicine and Dentistry
601 Elmwood Avenue, Box 697
Rochester, NY-14642
Tel: (585) 275-1810
E-mail: Lisa_DeLouise@urmc.rochester.edu

Supplementary Tables

Supplementary Table 1: List of Materials Used in the experiments

**Product**	**Company**	**Catalogue #**
Quantum Dots (QD)-Lipophilic	NN Labs	CZ-600-25
20 nm Silica Nanoparticles	Nanocomposix	ECP1011
50 nm Aminated Silica Nanoparticles	Nanocomposix	DAC1635
50 nm Silica Nanoparticles	Nanocomposix	STH0069
160 nm Silica Nanoparticles	Nanocomposix	DAC1239
400 nm Silica Nanoparticles	Nanocomposix	STH0018
Carbon Nanotubes (pore diameter 30nm, length 5-20 um, suspended in water 1 mg/ml)	Nano-lab	PD30L5-20-COOH
20 nm Gold Nanoparticles	Sigma Aldrich	753610-25ML
20 nm Silver Nanoparticles	Nanocomposix	AGCN20-25M
Titanium Dioxide (1% Mn Dopant)	Sigma Aldrich	677469-5G
Acetone (99.9% ACS grade)	JT Baker	9006-01
Polyethyleneimine Branched	Sigma Aldrich	408727-100ML
Methoxy Polyethylene glycol	Jenkem Technology	M-SH-5000
Glutathione	Calbiochem	3541
Dihydrolipoic Acid	Previously described (Zheng et al., J Biomed Nanotechnol, 2013, 9(3): 382-92	-
1-fluoro-2,4-dinitrobenzene	Sigma	D1529-10ML
Pure Olive Oil	Wegman's Brand	
3-pentadecylphenol	Tokyo Chemical Industry	P1202

Supplementary Table 2: QD Properties quantified using the Malvern Zetasizer at pH 6.7

Surface Coating	Zeta Potential (mV)	Hydrodynamic* Diameter (nm)
Glutathione GSH-QD	-23.0 ± 0.7 (Negative)	20.0 ± 1.5
Polyethyleneimine PEI-QD	+29.8 ± 0.3 (Positive)	26.7 ± 5.6
Polyethylene Glycol (Methoxy PEG-QD)	-3.0 ± 4.4 (Neutral)	26.9 ± 10.3
Dihydrolipoic Acid (DHLA-QD)	-26.2 ± 7.2 (Negative)	13.6 ± 0.71

* QD core/shell diameter ~6 nm

Supplementary Table 3: NP Properties quantified using the Malvern Zetasizer at pH 6.7

Particle	Size (nm)	Zeta Potential (mV)	PDI
Silica Nanosphere 20nm	32.71 +/- 2.07	-25.37 +/- 6.44	0.217
Silica Nanosphere 50nm	66.48 +/- 0.54	-45.67 +/- 0.59	0.076
Silica Nanosphere 50nm (aminated)	69.32 +/- 1.04	+17.74 +/- 13.10	0.132
Silica Nanosphere 160nm	184.93 +/- 0.76	-33.47 +/- 0.65	0.038
Silica Nanosphere 400nm	440.00 +/- 4.07	-65.97 +/- 0.70	0.005
Titanium Dioxide <100nm	556.40 +/- 33.36	-9.05 +/- 1.16	0.296
Silver Nanoparticle 20nm	73.89 +/- 10.25	-5.98 +/- 1.68	0.354
Gold Nanoparticle 20nm	69.32 +/- 27.33	-16.47 +/- 1.56	0.318

Supplementary Table 4: Concentration of Nanoparticles in the Co-Challenge Experiments

NP Type	Stock Concentration	Amount added to 0.2% DNFB in 4:1 Acetone:olive oil medium (500 µl)	Amount applied on the Co-challenge ear (20 µl)
**GSH-QD***	2.39 µM	24 µl	2.3 $\times 10^{-12}$ moles, ~2.8 µg
**DHLA-QD***	4.13 µM	14 µl	2.3 $\times 10^{-12}$ moles, ~2.8 µg
**PEI-QD***	9.72 µM	6 µl	2.3 $\times 10^{-12}$ moles, ~2.8 µg
**Methoxy PEG-QD***	2.75 µM	21 µl	2.3 $\times 10^{-12}$ moles, ~2.8 µg
**Organic QD-Lipophilic**	2.5 mg/ml	10 µl	1 µg
**Gold NP (AuNP 20nm)**	~7.2E11 particles/ml	25 µl	7.2E^{8} particles, ~0.06 ug**
**Carbon Nanotubes (CNT)**	1 mg/ml	25 µl	1 µg
**Titanium dioxide (TiO_2)**	10% by weight	0.05g	2 µg
**Silica NP 20nm**	10 mg/ml	10 µl	4 µg
**Silica NP 160 nm**	10 mg/ml	10 µl	4 µg
**Silica NP 400nm**	10 mg/ml	10 µl	4 µg
**Silica NP 50nm aminated**	10 mg/ml	10 µl	4 µg
**Silver NP 20 nm**	0.02 mg/ml	0.1 mg	4 µg

*QD MW ~1,210,000 per manufacture technical specification NNLabs, #CZ-600
** 8.08e-17 g /Au particle (20 nm) http://www.bbisolutions.com/molar-concentration-of-nanoparticles/

Supplementary Figure S1

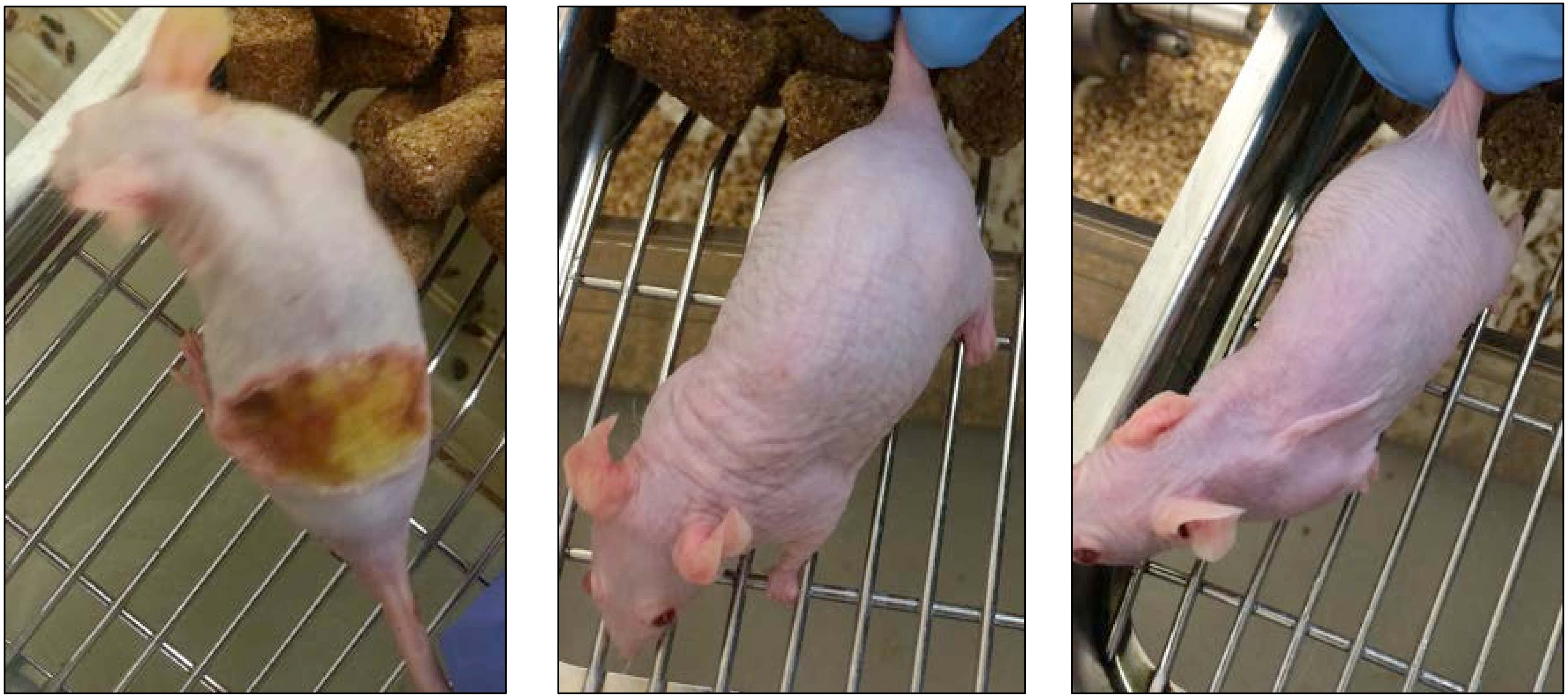

0.5% DNFB **0.05% DNFB** **0.025% DNFB**

Supplementary Figure S1: Gross representation of the mouse skin sensitized to 3 different DNFB doses

Mouse skin (dorsal back) was sensitized to 3 different concentrations of DNFB. 0.5% DNFB causes a chemical burn (eschar) on the skin which is not ideal for NP studies as the skin barrier is impaired. Titration studies indicated that a sensitization dose of 0.05% DNFB in an acetone/olive oil vehicle was sufficient to elicit the expected magnitude ear swelling response following challenge with 0.2% DNFB in our C57BL/6 hairless mice (**Figure S2**) without inducing an eschar that results when the mice are sensitized with the standard 0.5% DNFB dose.

Supplementary Figure S2

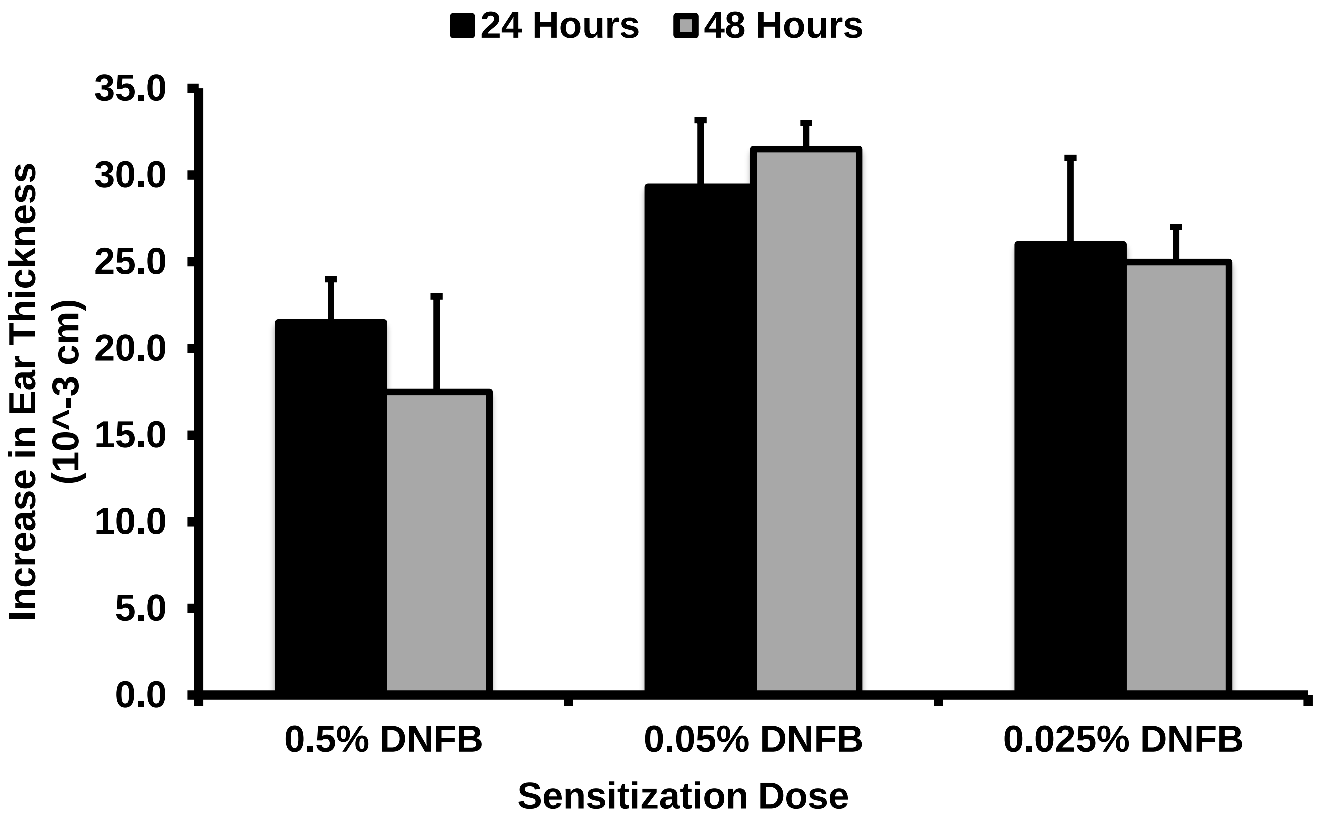

Supplementary Figure S2: Altering the sensitization dose does not alter the ear swelling response.

Mice were sensitized to 3 different doses-0.5%, 0.05% and 0.025% DNFB in 4:1 acetone: olive oil vehicle. The solution was pipetted on the mouse back (day 0). 5 days later the mice were challenged to 0.2% DNFB (Right ear) and vehicle alone (left ear). The ear swelling response was measured 24 and 48 hours after challenge and quantified with respect to the pre-measurement value. No significant differences were observed between the treatment groups at both 24 and 48 hours. Ears exhibited scab formation around 72 hours. N=3, 2 tailed t-Test, paired with unequal variances.

Supplementary Figure S3

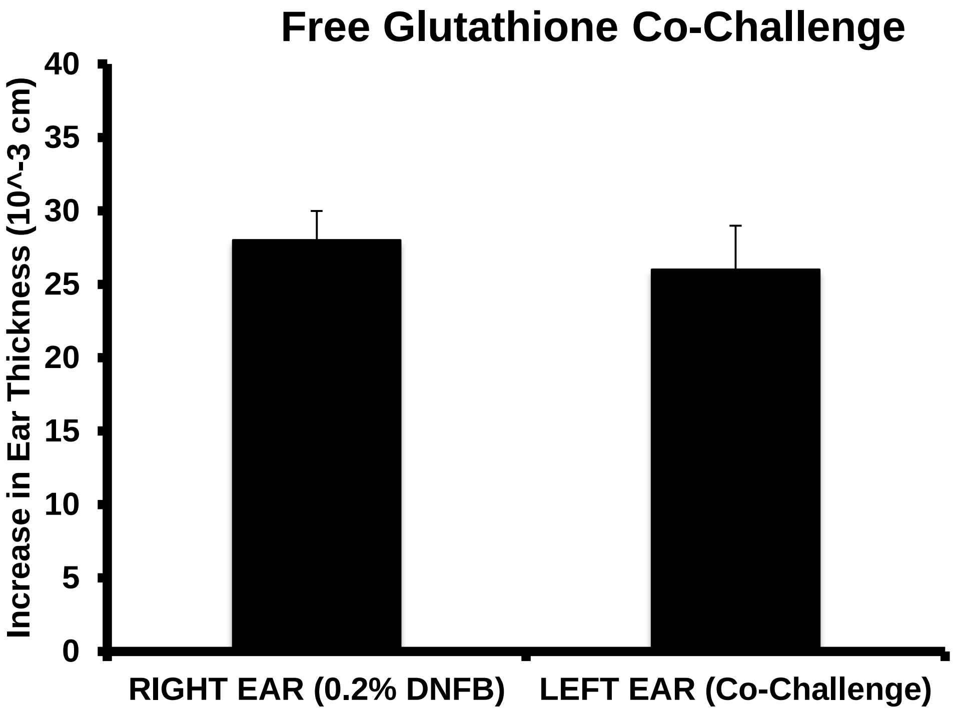

Supplementary Figure S3: Glutathione tested alone

The left ear was co-challenged with free glutathione plus 0.2% DNFB (left). The right ear was challenged with 0.2% DNFB only. Results show that free glutathione does not inhibit the swelling response compared to the 0.2% DNFB treated ear (right). Glutathione was weighed and added 10% by weight to the vehicle (0.05g GSH in 500 µl AOO, 0.325 M). A total 2 µg ($6.5x10^{-6}$ moles, GSH MW=307.3 g/mole) was applied to the co-challenge ear. This dose of free GSH is 1000X more than is estimated to be applied using the GSH-QD assuming that each QD had ~1000 GSH molecules tethered to the surface (**Table S4**).

Supplementary Figure S4a

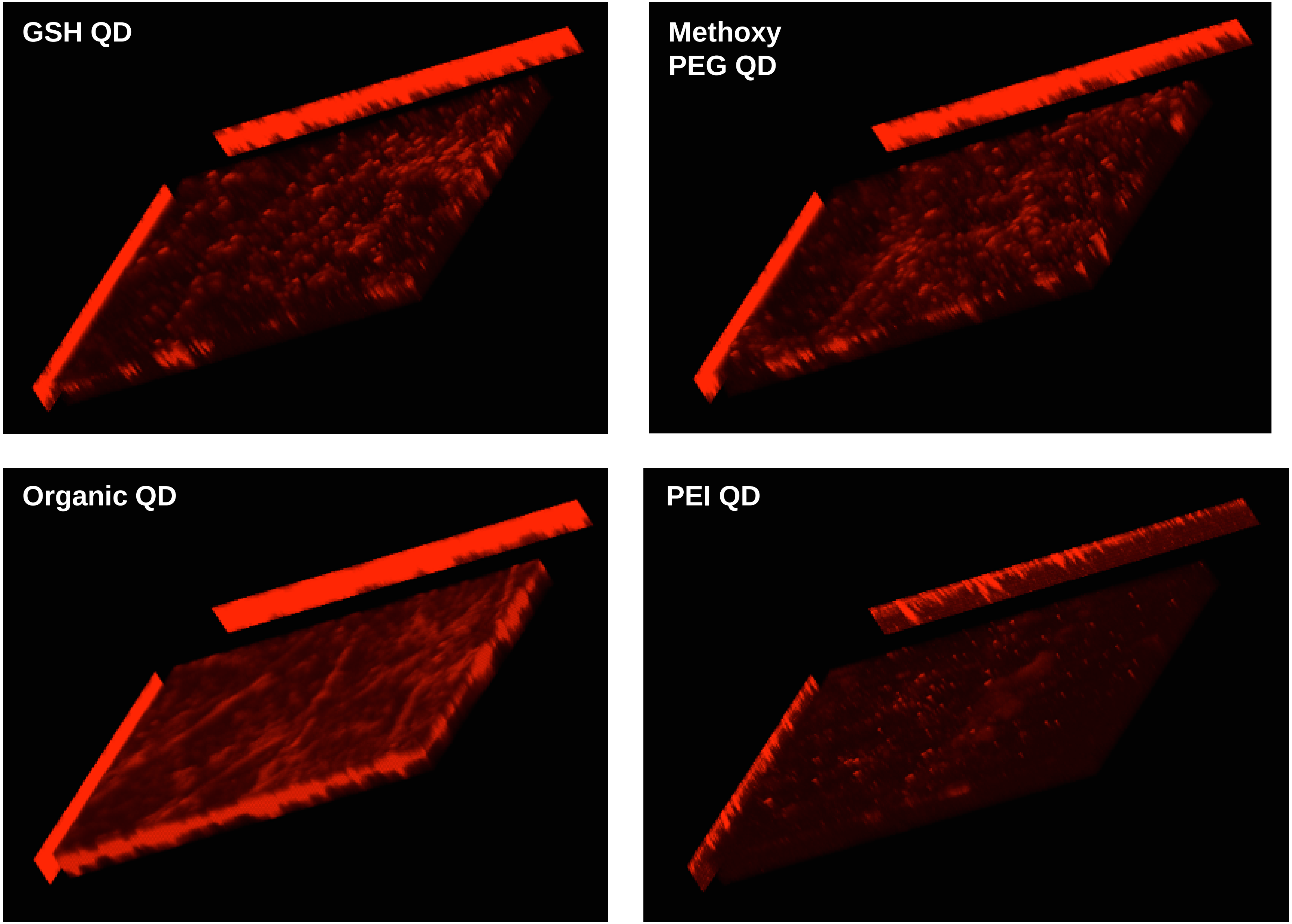

Supplementary Figures S4b & S4c

4b

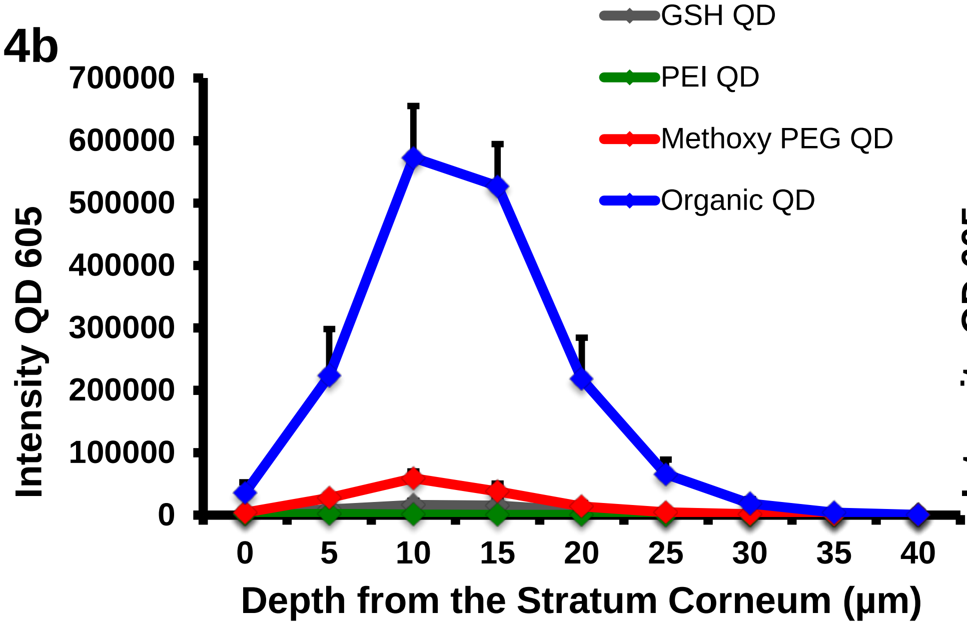

4b-Inset

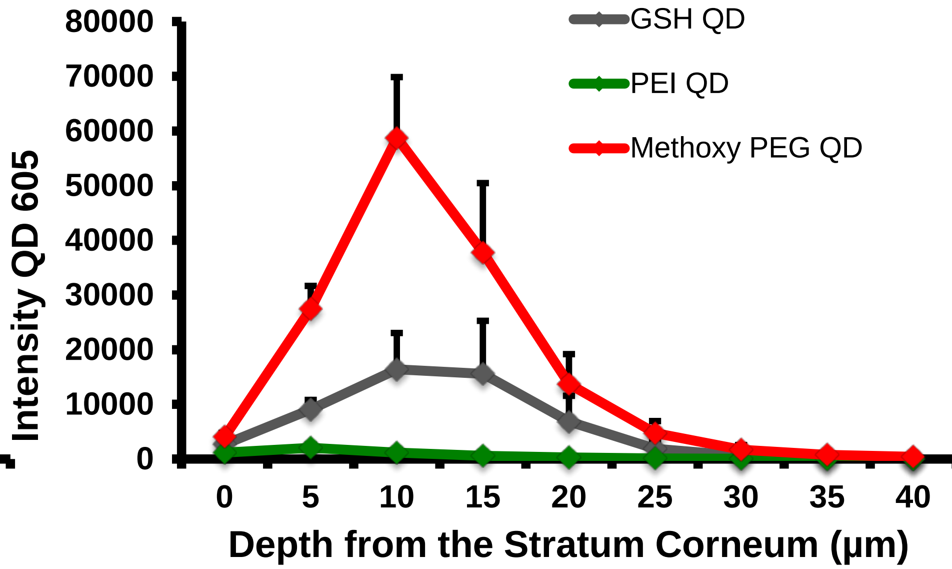

4c

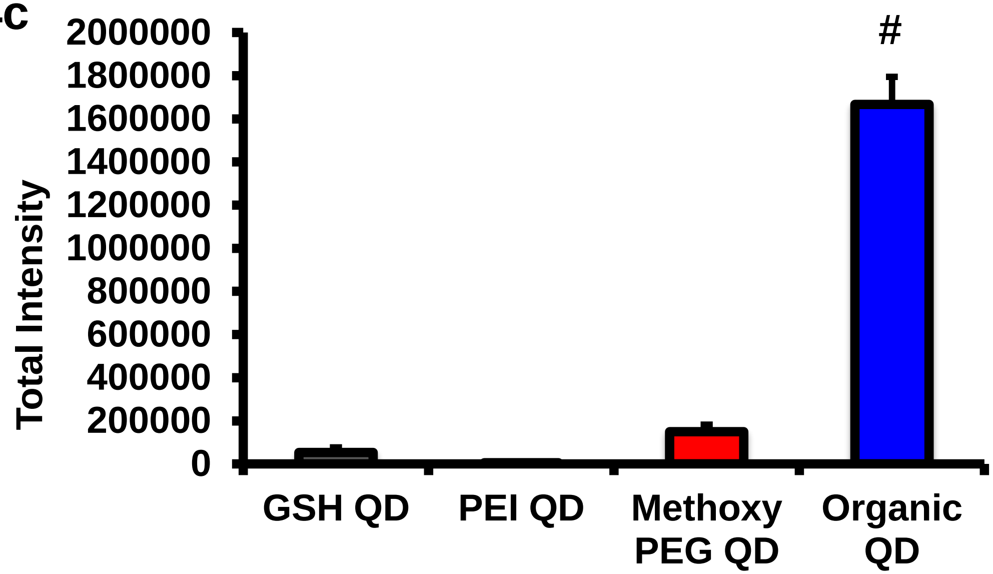

4c-Inset

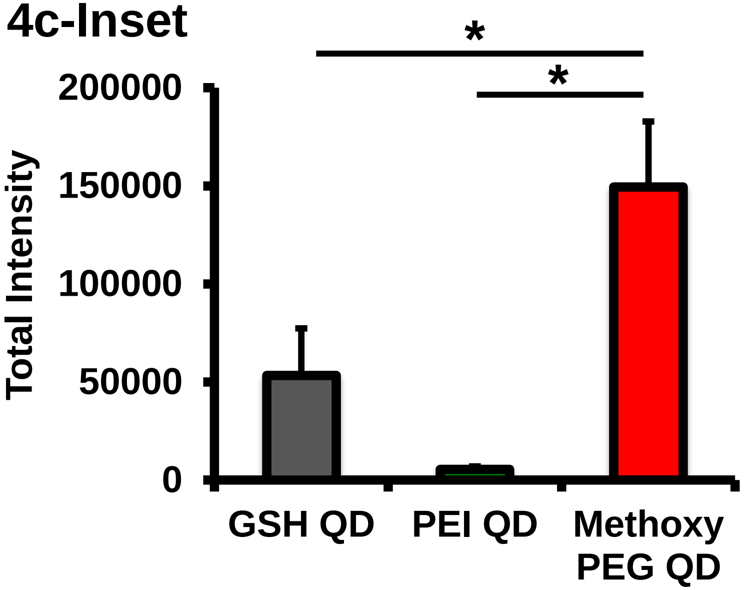

Figure S4: Quantification of *ex vivo* penetration of quantum dots in mouse skin using confocal microscopy

GSH, PEI, methoxy PEG and organic QDs were applied to mouse skin *ex vivo* in an acetone/olive oil vehicle for 24 hours. The samples were wiped with PBS to remove QDs on the skin surface and then imaged using confocal microscopy to detect QD fluorescence in the skin from 0 µm (stratum corneum) to 40 µm deep into the epidermis. The stacks were processed using ImageJ. **(4a)** Side profile view of image stacks shows that GSH, methoxy PEG and organic QDs penetrate more uniformly and deeper into the skin as compared to PEI QDs. PEI-QDs are present in the stratum corneum and accumulation in hair follicles is prominent. **(4b)** Plot shows the QD intensity integrated from 0-40 µm. For all treatment groups the QDs are concentrated in the region between 0-25 µm. The organic QDs accumulate in skin to a greater extent and there is minimal detection of PEI-QD. **(4c)** The bar plot shows the overall QD presence in the treatment groups with organic QDs showing the highest overall retention in skin and PEI-QD the lowest.

*$p<0.05$, 2 tailed t-Test, unpaired with unequal variances

# $p<0.05$, 2 tailed t-Test, unpaired with unequal variances (organic QD group significant wrt to all other test groups), N=5.

Supplementary Figure S5

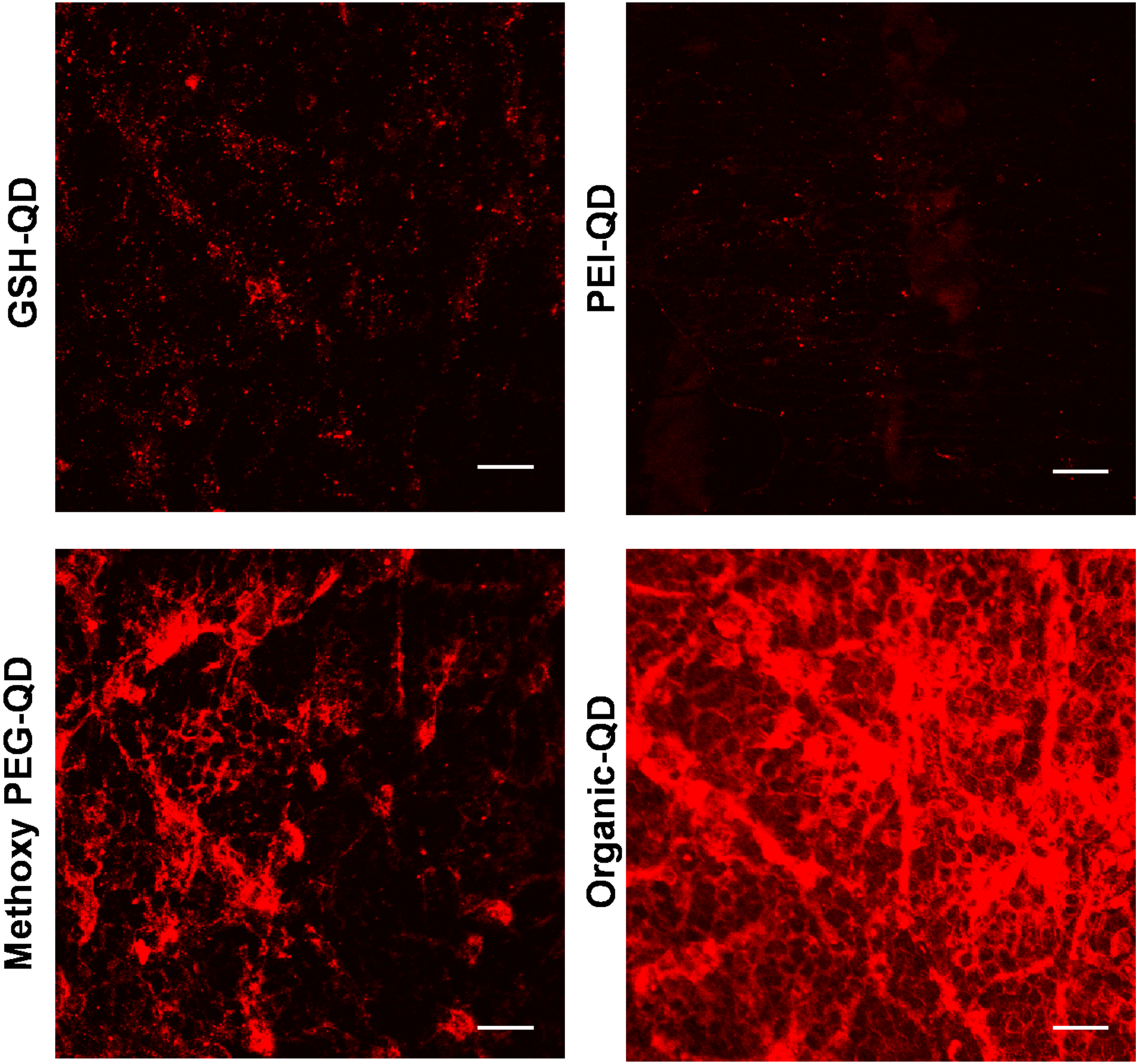

Supplementary Figure S5: Top down view of skin sections on the confocal microscope at 10 μm depth from the stratum corneum

Ex vivo mouse skin exposed to QDs coated with different ligands was imaged using confocal microscopy. Organic-QDs are retained in skin to a much greater extent compared to all other treatment groups. PEI-QDs penetrate the least through the stratum corneum. Scale Bar=5 μm.

Supplementary Figure S6

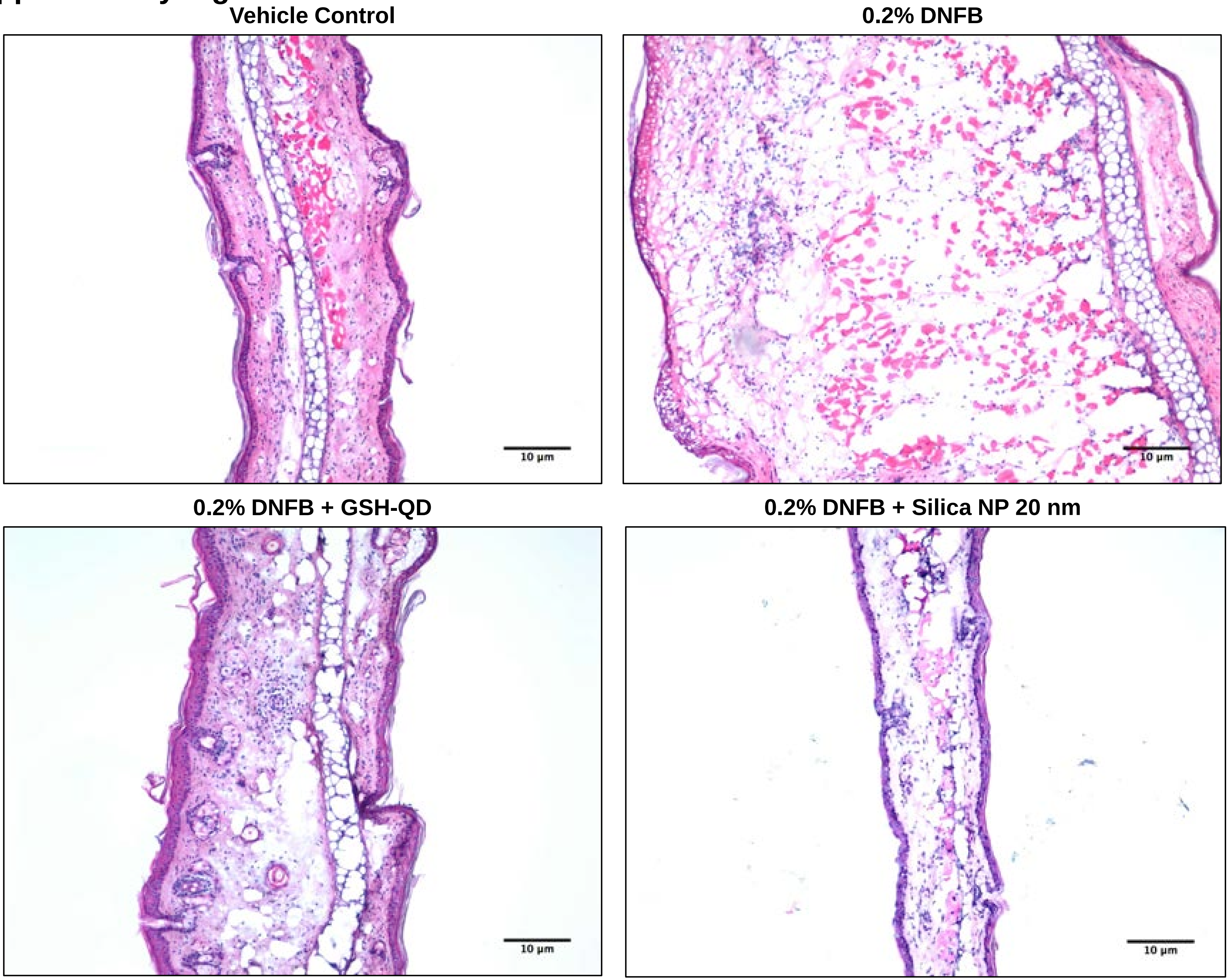

Supplementary Figure S6

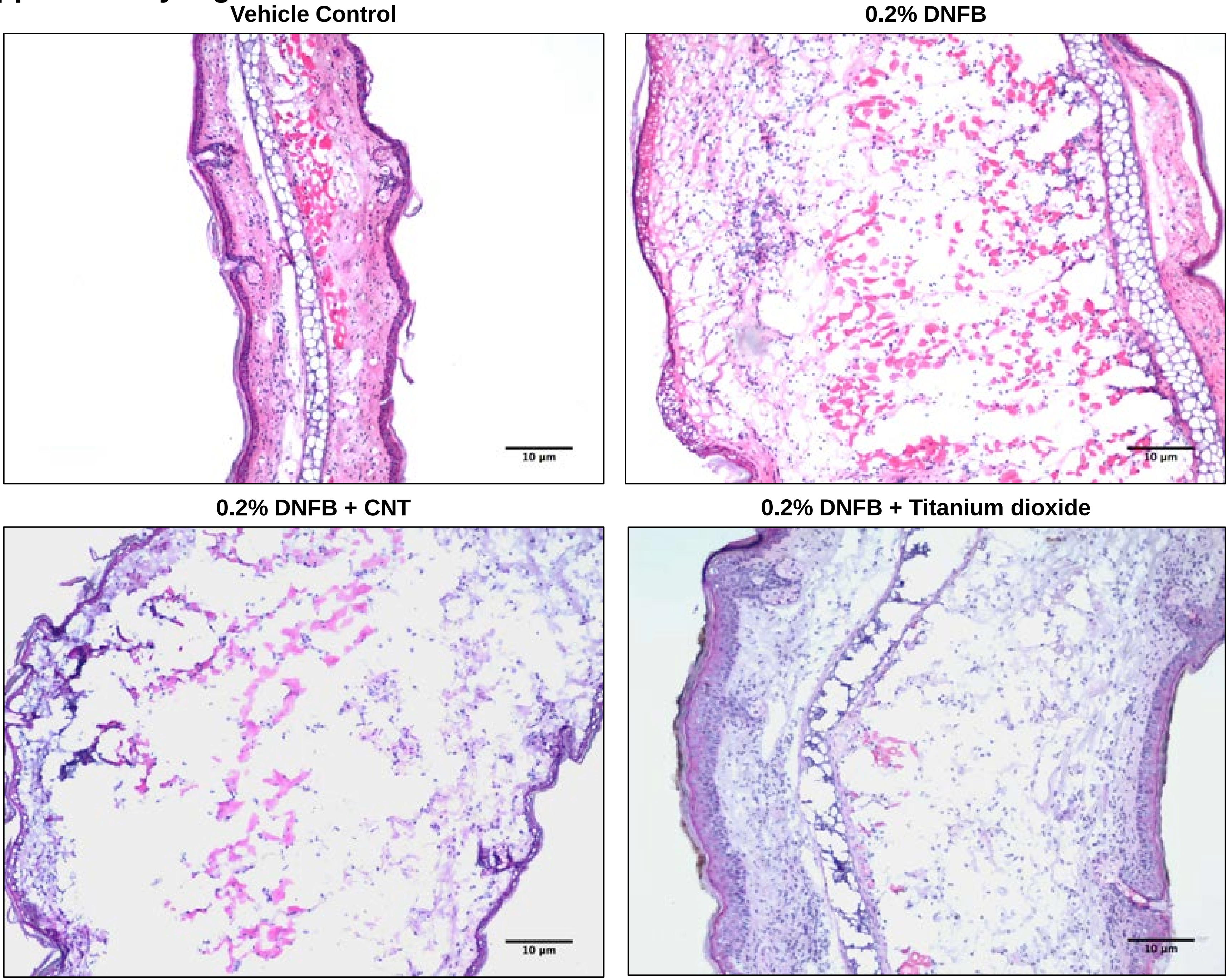

Supplementary Figure S6: Ear Sections stained with H&E (Hematoxylin and Eosin Stain)

H&E sections from mouse ears show inhibition of the ear swelling response in the case of GSH-QDs and Silica NP (20nm) compared to vehicle ear. There are fewer cell infiltrates observed in the these ear sections. A huge swelling response is observed in the DNFB, DNFB+CNT, DNFB+ TiO_2 treated ears. A large number of cell infiltrates can be observed in these sections.

Supplementary Figure S7

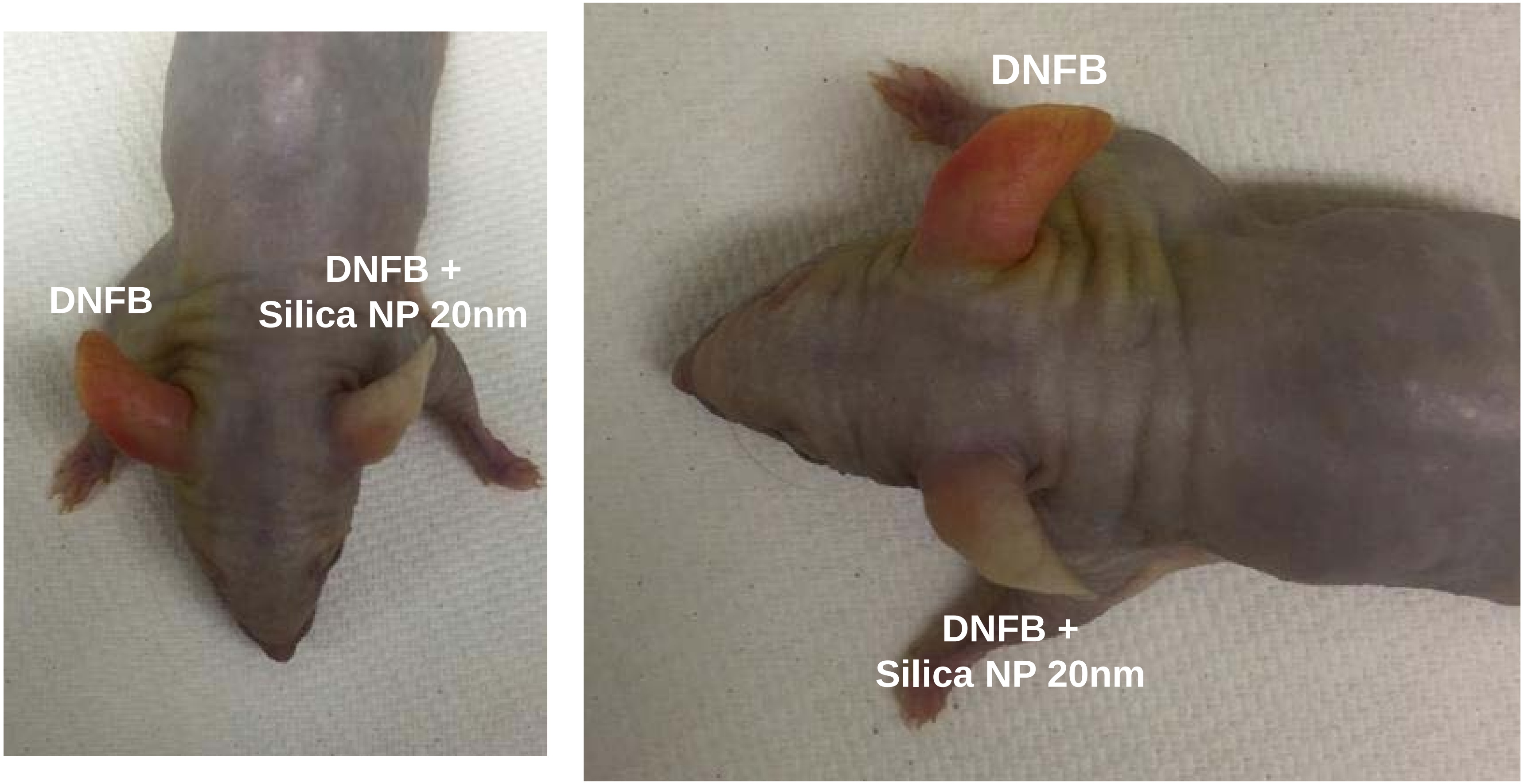

Supplementary Figure S7: Gross Representation of the Ear Swelling Response when co-challenged with Silica NP 20nm

Supplementary Figure S8

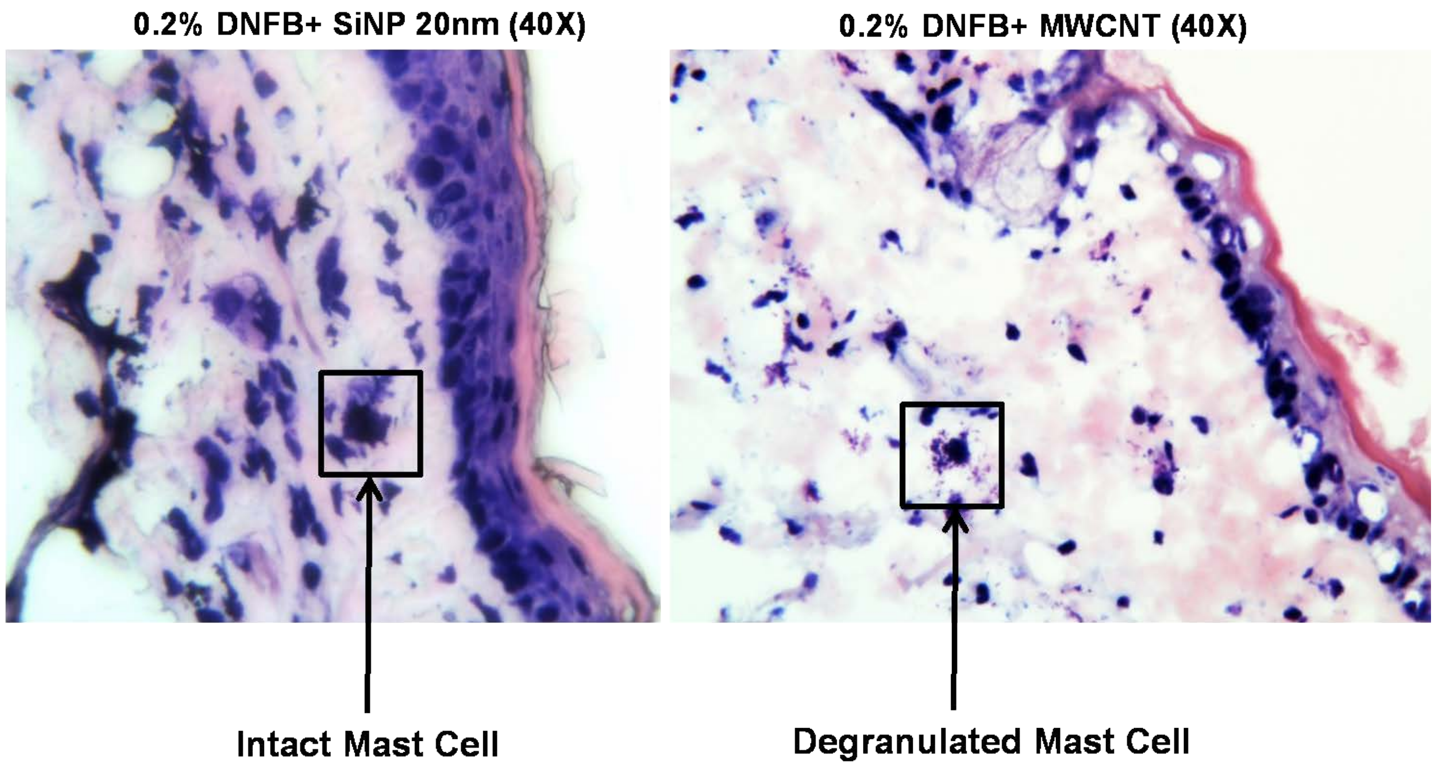

Figure S8. Quantification of intact vs. degranulated mast cells in tissue stained with Geimsa.
Mast cells were quantified at 40X magnification in individual tissue slices treated with various DNFB+NP combinations. Shown in the figure above are representative examples of intact and degranulated mast cells (Geimsa stain). Dermatopathologist, Dr. Glynis Scott from Dermatology (URMC), verified the count.
